# Supplementary material for: The causal relationship between the human gut microbiota and pyogenic arthritis: a Mendelian randomization study
Source: Front Cell Infect Microbiol. 2024 Nov 26;14:1452480. doi: 10.3389/fcimb.2024.1452480 (PMC11629706; doi:10.3389/fcimb.2024.1452480)
Supplement: Supplementary file 1 [file DataSheet1.zip › mendelian test/ebi-a-GCST90027619.csv_forest.pdf]

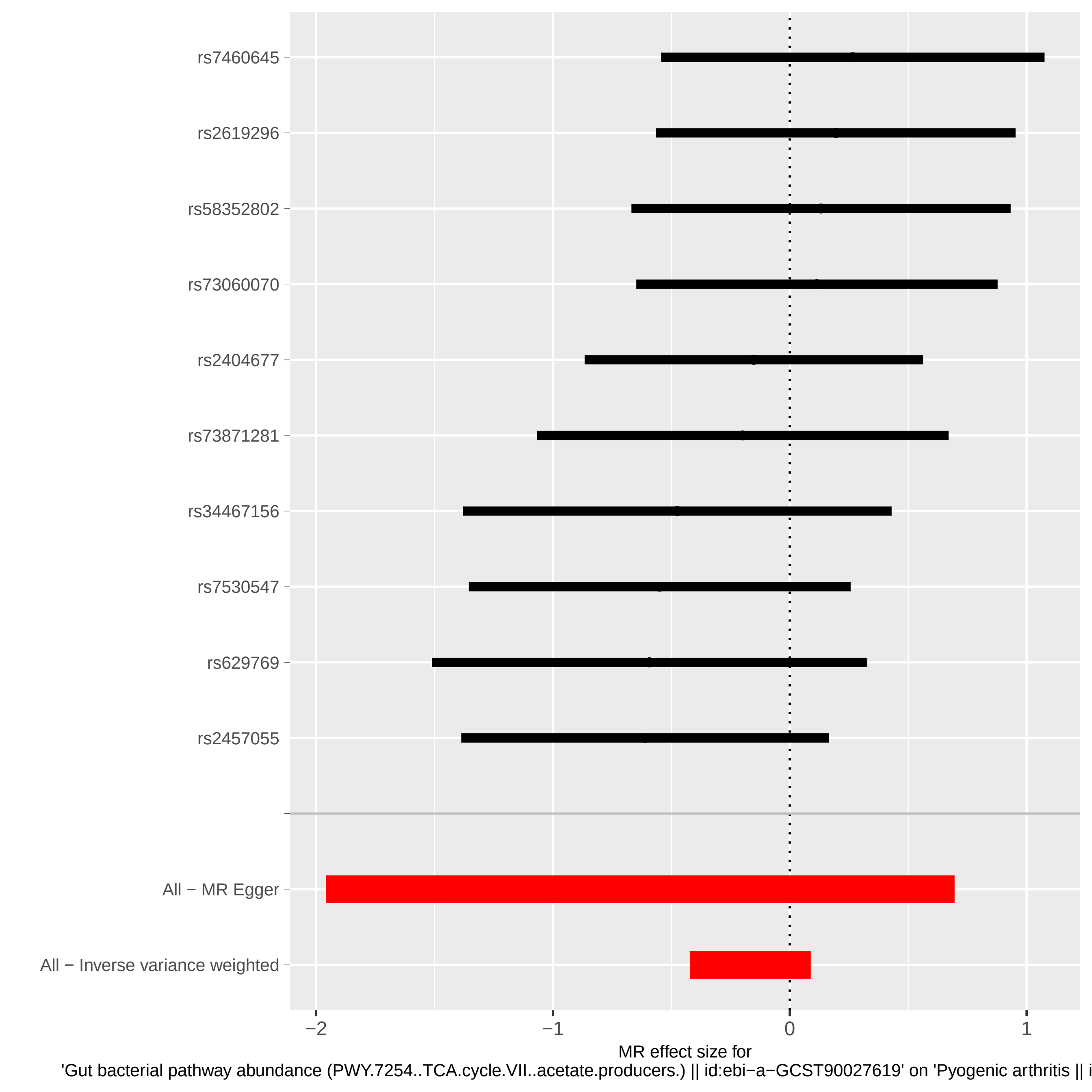

rs7460645
rs2619296
rs58352802
rs73060070
rs2404677
rs73871281
rs34467156
rs7530547
rs629769
rs2457055
All − MR Egger
All − Inverse variance weighted
−2
−1
0
1
MR effect size for
'Gut bacterial pathway abundance (PWY.7254..TCA.cycle.VII..acetate.producers.) || id:ebi−a−GCST90027619' on 'Pyogenic arthritis || i
